# Intein-mediated recombinant expression of monomeric B22Asp desB30 insulin

**DOI:** 10.1186/s12896-020-0598-3

**Published:** 2020-01-09

**Authors:** Minmin Zhang, Yunlong Zhang, Bingnan Wu, Yanhao Peng, Altaf Ahmed Simair, Geoffery W. Siegel, Changrui Lu, Ting Chen

**Affiliations:** 1 Key Laboratory of Science and Technology of Eco-Textiles, Ministry of Education, College of Chemistry, Chemical Engineering and Biotechnology, Donghua University, 2999 North Ren Min Rd, Shanghai, 201620 China; 2 Department of Orthopaedic Surgery, Musculoskeletal Oncology Division, University of Michigan Medical School, Ann Arbor, MI 10 USA

**Keywords:** Intein, B22D desB30 insulin, Self-cleavage, Optimization

## Abstract

**Background:**

Insulin controls hyperglycemia caused by diabetes, and virtually all treatments require exogenous insulin. However, the product’s extensive post-translational modifications have hindered the manufacture of recombinant insulin.

**Result:**

Here we report a novel production method for a monomeric B22Asp desB30 insulin analog (B22D desB30 insulin). Its precursor, DPIP, is fused to an N-terminal chitin-binding domain and intein self-cleavage tag. The fusion protein is expressed and purified from *E. coli* and immobilized on chitin resins. DPIP is then released using an optimized pH shift and converted to mature insulin via trypsin digest. The resulting product appears monomeric, > 90% pure and devoid of any exogenous enzyme.

**Conclusion:**

Thus, biologically active insulin analog can be efficiently produced in bacteria and potentially applicable in the treatment of human diabetes.

## Background

Diabetes mellitus in human causes elevated blood sugar levels for a prolonged period of time [1]. If untreated, the disease may progress into many life-threatening complications, like kidney disease, blindness, and amputations [2]. Virtually all patients require regular intake of exogenous insulin, which lowers their blood glucose concentration. Studies suggest that the disease affects hundreds of millions of people, with an annual cost rapidly approaching one trillion dollars [3].

Since 1977, production of animal insulin has gradually exited the market as recombinant human insulin emerged [4]. However, since the human insulin monomers readily aggregate into multimers, the recombinant wild type human insulin displays a delay in treating hyperglycemia [5]. Therefore, its monomeric analogs, termed fast-acting insulins, are developed and clinically proven to match human endogenously produced insulin [4, 6]. However, both the manufacturing process and composition of these patented insulin analogs are closely guarded commercial secrets. Due to the increasing diabetic population and rapidly rising cost, the market urgently requires the development of low-cost, fast-acting insulin analogs, especially in the underdeveloped and developing nations.

Here we report recombinant production of a fast-acting insulin analog, termed B22D desB30, matured from a single-chain precursor. In vivo assays have shown that analogs of B22D desB30 stays monomeric and exhibits 30–40% activity, compared to the human endogenous counterpart [7–9]. However, structural changes that prevented multimerization also possibly destabilized its overall folding and hence made production difficult [10]. Therefore, we opted to recombinantly express and purify the B22D desB30 precursor by protein splicing, which yielded single chain insulin precursor (B22D-PIP or DPIP).

Protein splicing involves the precise excision of an intein from a primary translation product concomitant with the ligation of the exteins via a peptide bond [11]. This autocatalytic process occurs post-translationally, without other enzymes or any cofactors [12, 13]. Several biotechnological applications explore the splicing properties of inteins, including protein purification, peptide cyclization and protein labeling [14–16]. Among them, the intein mediated purification with an affinity chitin-binding tag (two Intein or IMPACT-TWIN by New England Biolabs) provides a low-cost, convenient system, in which the target protein is immobilized by a single-step affinity enrichment and collected by intein self-cleavage that removes the affinity tag [17, 18]. Using this process, we obtained the native protein without any affinity tag or exogenous proteases. Subsequent tryptic digestion produced pure, monomeric B22D des30 insulin analog.

## Results and discussion

### CBD-intein1-DPIP fusion protein expresses in the inclusion body

**Fig. 1:**
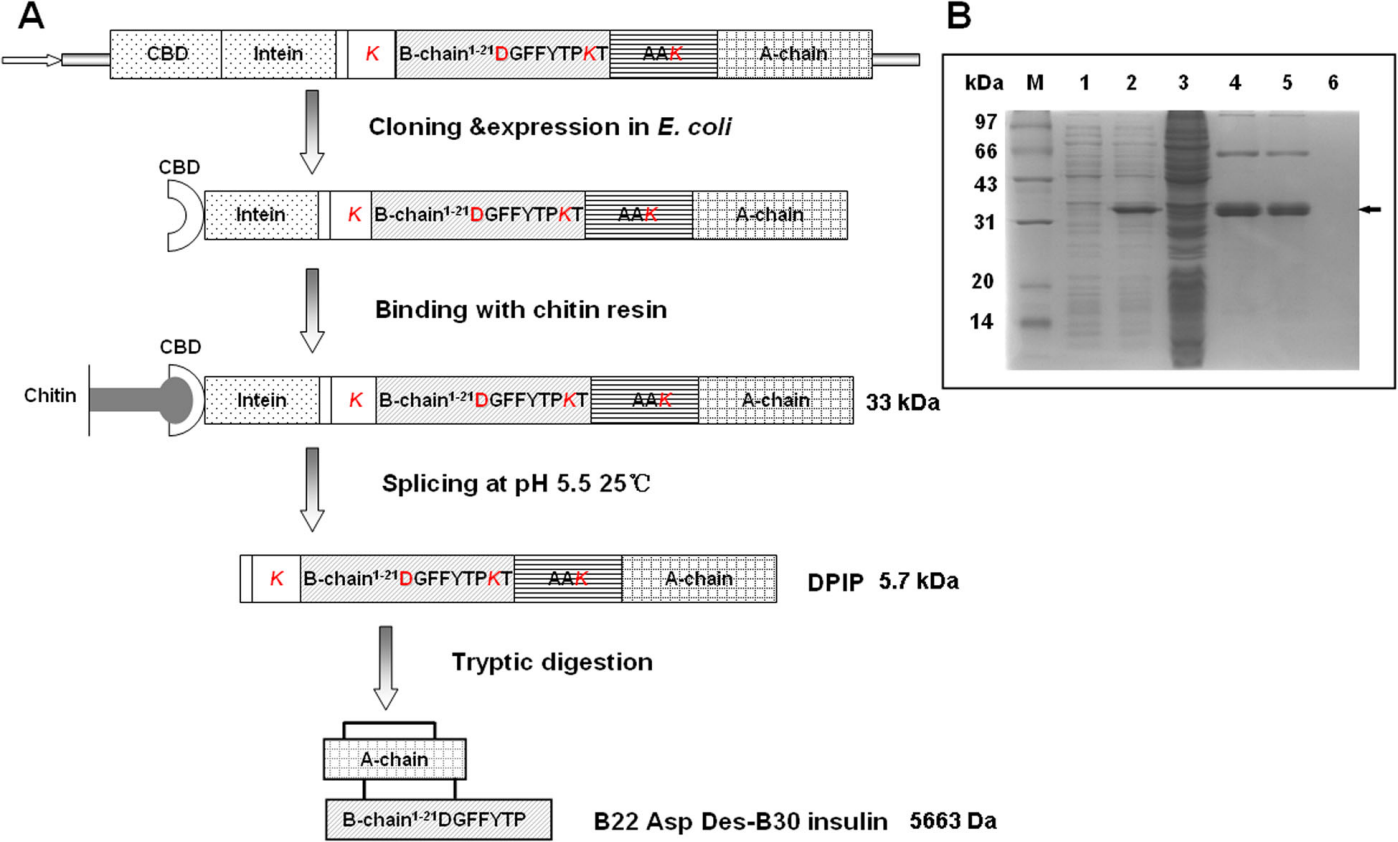
Purification of DPIP by the IMPACT-TWIN system. **a**: Scheme of the protein expression and purification. **b**: *E. coli* strain BL21 (DE3) transformed with pTWIN1-DPIP was cultured, induced with IPTG and fractionated to purify the recombinant fusion protein. Fractions were analyzed by 12% SDS–PAGE. M: MW marker (97, 66, 43, 31, 24, 14 kDa), 1: E coli lysate before induction, 2: lysate after induction, 3: soluble fraction after induction. 4: redissolved inclusion body before dialysis, 5: redissolved fraction after dialysis, 6: redissolved, dialyzed fraction flow through. An arrow marks the full-length fusion protein

The recombinant B22D desB30 produced in this study is converted from a precursor (DPIP) fused to a CBD-intein tag. Fig. 1a shows the recombinant expression scheme of B22D desB30 insulin analog. The construct CBD-Intein-DPIP was confirmed by sequencing and all expression was carried out at similar conditions.

The recombinant fusion protein is consistently expressed at a high level (Fig. 1b, lane 2). Most likely the target protein collects in the inclusion body and is insoluble. The fusion protein expresses well in the inclusion bodies and was insoluble, consistent with precious studies [20, 21]. After collecting the pellet after cell lysis, the fraction that redissolved after urea treatment is enriched with the 36 kDa target protein (Fig. 1b, lane 4). Solubilizing the inclusion bodies with urea allows the ready purification of the fusion protein to near homogeneity. Subsequent affinity chromatography traps nearly all renatured fusion protein on the chitin matrix. All renatured protein binds to the column after dialysis, which suggests that the re-naturation process correctly restored native protein folding (Fig. 1b, lane 5). The CBD-intein tag also allows a pH-induced auto-cleavage at the N-terminus of the insulin precursor, releasing the peptide without exogenous protease or tags.

Since intein cleavage is sensitive to both temperature and pH, we aim to fine-tune the reaction condition to maximize purification efficiency. This allows the most efficient release of the target DPIP with simple temperature and pH shift. By carrying out the cleavage reaction at three different temperatures (10 °C, 25 °C and 30 °C) and eight pH conditions (4.5 to 8.0, 0.5 interval), our study shows that the intein self-cleave reaction is strictly temperature- and pH-dependent.

**Fig. 2:**
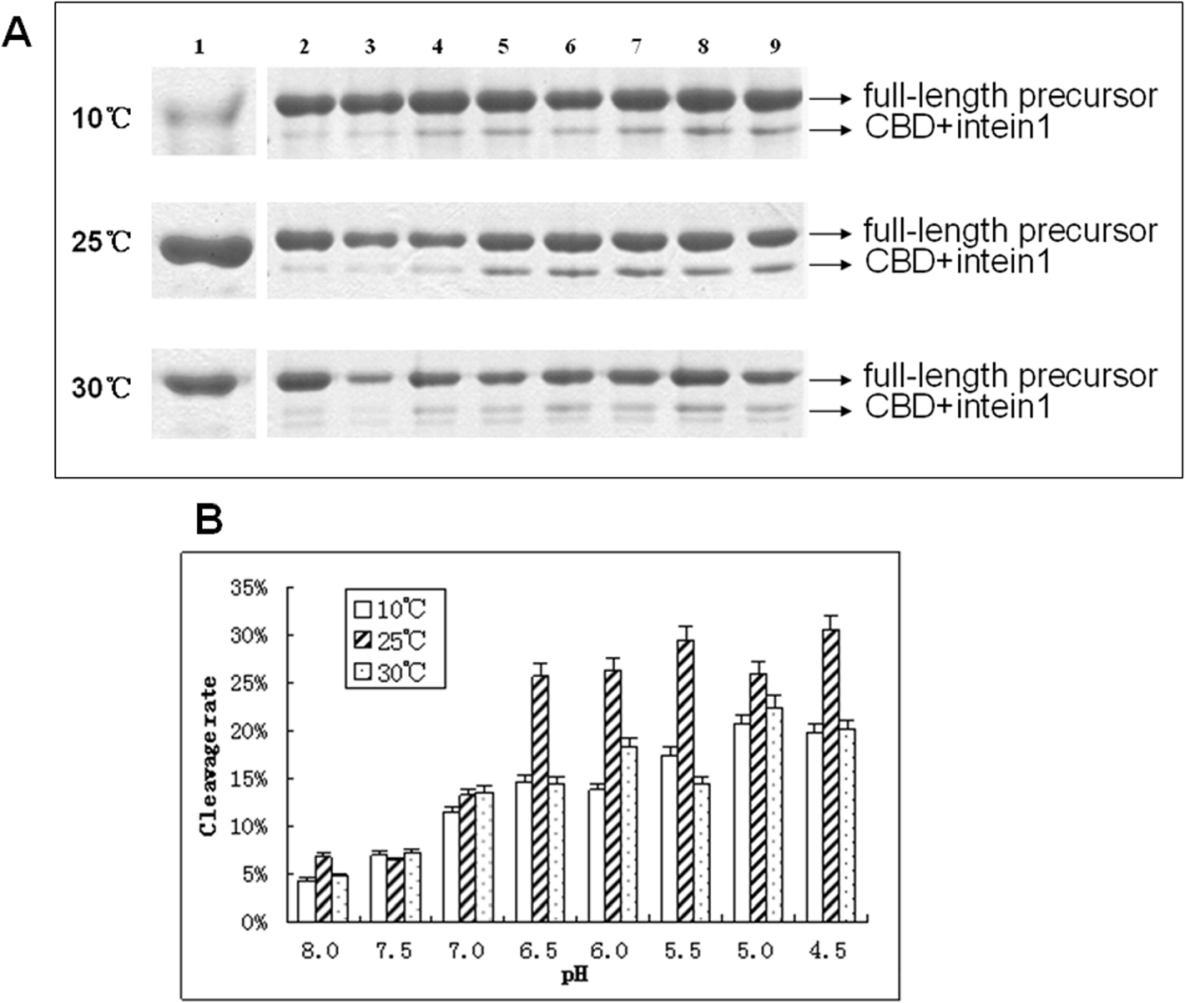
Both temperature and pH affect CI-DPIP cleavage in vitro. **a**: SDS-PAGE analysis of cleavage products after incubation at different pH and temperature. Lane 1: Input before cleavage reaction, Lanes 2–9: the resin after cleaving by TGE buffer with pH 8.0, 7.5, 7.0, 6.5, 6.0, 5.5, 5.0, 4.5. **b**: quantified cleavage ratio. All assays were carried out in triplicate and the average was used in this study; the error bars show standard deviations

During optimization of the pH-induced intein self-cleavage, we discovered that lowering the pH below physiological conditions aided the cleavage process. Fig. 2a shows proteins retained by the chitin column by SDS-PAGE after reaction at various temperature and pH. Lane 1 shows that the fusion protein (CI-DPIP, 36 kDa, marked by the top arrow) bound to the column before cleavage. Lanes 2 through 8 indicates that one of the products (CBD + intein1, 31 kDa, marked by the lower arrow), which also tightly associates with the column, decreases in intensities with the increase of pH (quantified in Fig. 2b). In general, we observed an increase in reaction activity (almost 100%) at lower pHs compared to physiological pH. This result is somewhat unexpected given the proposed mechanism for C-terminal intein cleavage involves deprotonation of the amino group [22].

This observation coincides with previous results [19, 23] in which the intein C-terminal cleavage rate increased when the pH was lowered to 6.0. Therefore, we speculate that this phenomenon maybe general, and acidic conditions may benefit the self-cleavage reaction of other intein fusion proteins. Our data suggest that this particular C-terminal intein cleavage is perhaps catalyzed by a general acid (hydronium) via alternative mechanisms. However, at extremely acidic environments (pH < 5.5), we suspect that DPIP (pI = 4.9) may become unstable or insoluble. Therefore, we determine that the optimal cleavage pH for CI-DPIP is 5.5. Previous studies suggest that intein may undergo different reaction mechanisms at varying pHs. However, no previous study has investigated intein cleavage conditions below pH 6.0 either experimentally or theoretically. Theoretical approaches proposed that at lower pH (6.0), especially below the pKa of histidine (6.04), the C-terminal cleavage reaction may take a different reaction path and increase its reaction rate [24]. Their result suggests that N-protonation of the scissile peptide bond likely starts the C-terminal cleavage reaction, in contrast to a nucleophilic side chain cyclization mechanism proposed at physiological pH [22]. Our results support the acid-catalyzed reaction mechanism hypothesis at low pHs.

While having very little effect on cleavage efficiency at higher pHs, temperature significantly increases the reaction rate at pH 6.5 and lower. Among all three temperature conditions tested, 25 °C shows, on average, 40% higher activity than 10 °C and 30 °C at pH 6.5–4.5. Although higher temperatures usually produce higher reaction rates, CI-DPIP cleaved inefficiently at 30 °C, similar to 10 °C. This result suggests that the intein structure in this construct may be unstable and the active site perturbed at 30 °C.

Our above data suggest that a simple pH change at 25 °C can induce the release of tag-less DPIP from the recombinant protein. At pH 7.5–8.0, the cleavage rate is the slowest (below 5%), which means that the precursor can be stored safely under these conditions. Once shifted to 25 °C and pH 5.5, the intein self-cleavage reaction rate constant increased significantly to 30%. Therefore, we conclude that the optimal temperature and pH for CI-DPIP cleavage in this study is 25 °C and pH 5.5. By shifting the buffer pH from 8.0 to 5.5, we can release pure insulin analog precursor DPIP from the column.

### Intein cleavage increases over two days

**Fig. 3:**
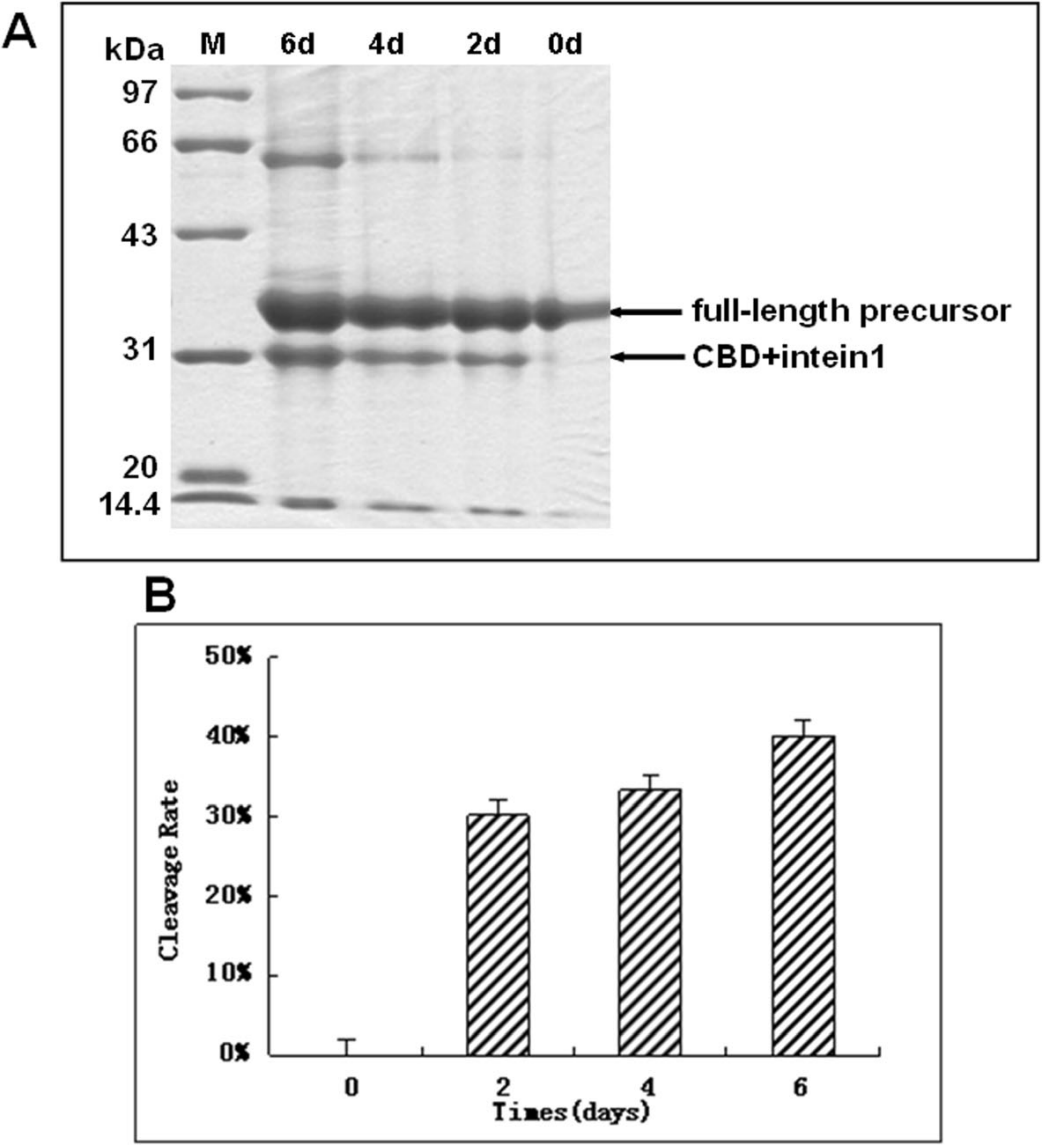
Rate of CI-DPIP cleavage increases then slows down after two days. **a**: SDS-PAGE analysis of cleavage with different incubation time. Lanes 1–3 indicate resin after incubation of 6 days, 4 days and 2 days, respectively, and lane 4 indicates the input precursor without any cleavage (0d). Since the precursor is chemically stable in solution, so Lane 4 shows no CBD + intein fraction, M: molecular weight markers. **b**: Quantified cleavage ratio. All assays were carried out in triplicate and the average was used in this study; the error bars show standard deviations

After determining the optimal temperature and pH for the intein cleavage reaction, we set to improve the yield of DPIP by investigating different reaction durations. We had explored reaction time on the self-cleavage of intein by comparing the resin samples after 2, 4 and 6 days of cleavage. During a six-day incubation, resin samples were collected separately every other day. Subsequent SDS-PAGE (Fig. 3a) and band analysis (Fig. 3b) showed that the reaction rate increases drastically over the first 2 days and leveled off after that. Although small amounts of product continued to form over the next four days, the amount of additional product cannot justify the extra time required and the risk of degradation. Therefore, two days was chosen as the optimal reaction time.

### Urea inhibits intein self-cleavage

**Fig. 4:**
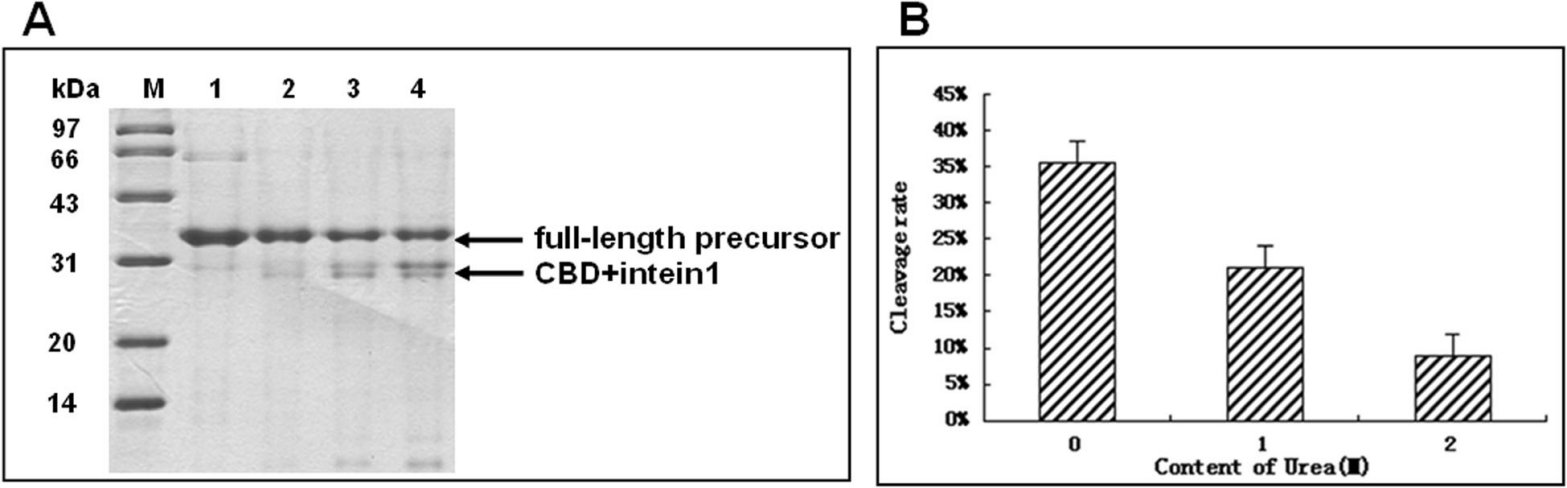
Urea inhibits intein self-cleavage of CI-DPIP. **a**: SDS-PAGE analysis of cleavage in the buffer with different concentrations of urea. M: Molecular weight markers. Lane 1: input. Lanes 2 and 3: resin after cleavage by 2 M and 1 M urea-containing buffers. 4: resin after cleavage without urea. **b**: calculated cleavage ratio of every cleavage reaction. All the cleaving reaction was completed in the TGE buffer (pH 5.5) at 25 °C for 2 days

Our study shows that the CI-DPIP construct is sensitive to urea. Since previous findings show that low concentrations of urea may increase the intein cleavage ratio [20], we decided to investigate the effect of urea on CI-DPIP self-cleavage. We tested two urea cleavage conditions (1 M and 2 M) against the no urea TGE buffer, and the results show that even one-molar urea can reduce reaction efficiency by 43% (Fig. 4). At two-molar urea concentration, the reaction efficiency dropped to below 10%. This result suggests that our CI-DPIP fusion protein is more sensitive to folding, and slight perturbation abolishes the intein activity. This is also consistent with our temperature study that higher temperatures adversely affect the cleavage reaction, presumably due to misfolding at higher temperatures. It is possible that urea and higher temperate adversely affect CI-DPIP splicing by similar mechanisms.

### DPIP precursor generates pure B22D desB30 insulin analog

**Fig. 5:**
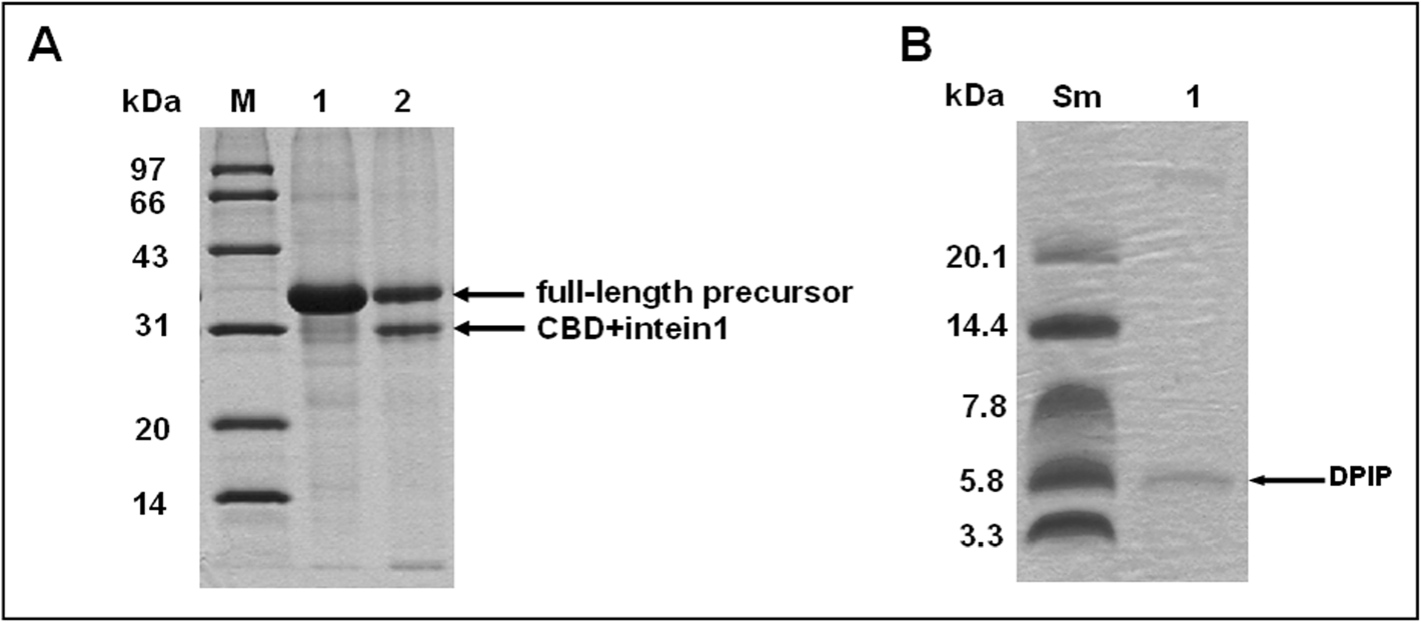
DPIP is released from CI-DPIP after intein cleavage. **a**: Cleavage of the DPIP fusion protein under the optimized condition. M: MW marker (97, 66, 43, 31, 24, 14 kDa). Lane 1: the chitin resin after protein binding. Lane 2: the resin after intein cleavage. The fractions were analyzed by 12% SDS-PAGE. **b**: DPIP eluted from resin. M: MW marker (20.1, 14.4, 7.8, 5.8, 3.3 kDa). Lane 1: DPIP eluate. The fractions were analyzed by 16.5% Tricine-SDS-PAGE

Since the smaller size of DPIP (MW = 5.7 kDa) does not allow accurate analysis on SDS-PAGE (Fig. 5a), 16.5% tricine gels were required to visualize and analyze the fragment (Fig. 5b). Our result clearly identifies the pure DPIP eluted from the chitin matrix using conditions described above (Table 1).

**Table 1:** Summary of DPIP Purification

c	Total Protien^a^(mg)	Target protein^b^(mg)	Approximate purity^c^(%)	Yield^d^(%)
Cell lysis	200.33	133.82 (CBD-intein-DPIP)	66.8	100
Refolding of fusion protein	156.26	116.73 (CBD-intein-DPIP)	74.7	87.2
Chitin column and intein-mediated cleavage	0.621	0.548 (DPIP)	88.3	2.4

One liter of culture contained about 3.2 g wet weight cells

^a^: Total protein was quantified by Bradford assay

^b^: The amount of fusion protein was calculated based on the following formula: target protein = total protein amount x approximate purity

^c^: The purities of fusion protein were determined by densitometry analysis of their corresponding SDS gel bands

^d^: The relative low recovery of DPIP was partially due to the low molecular weight of DPIP (6 kDa) compared to the fusion precursor CBD-intein-DPIP (33 kDa)

**Fig. 6:**
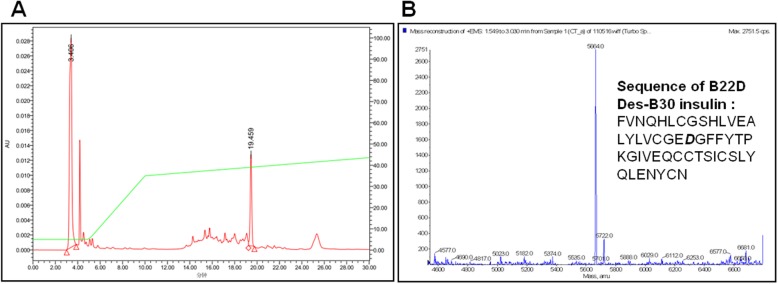
Purification and mass spectroscopy confirm B22D desb30 insulin. **a**: HPLC of B22D desB30 insulin. The UV absorption was detected at 280 nm. Conditions: equipment, HPLC Waters 600 (Waters, USA). Column: Kromasil C8 column (4.6 × 250 mm). Mobile phase: buffer G (0.1% TFA), buffer H (90% HPLC grade acetonitrile in H2O, 0.08% TFA). Gradient: 35–50% buffer B in 30 min. Flow rate: 1 ml/min. Injected volume: 200 μl. **b**: mass spectroscopy analysis of B22D desB30 insulin. The determined MW is 5664.0 Da

To obtain the final folded insulin monomer, DPIP was subjected to trypsin digest. This step releases A-chain and B-chain from DPIP, removing the linker peptides (GRAK at the N-terminus and AAK between A-chain and B-chain) (Fig. 1a). Subsequently, two disulfide bonds spontaneously link A-chain and B-chain to form the final monomeric product. This insulin analog migrated as a single peak at 19.5 min during our HPLC purification (Fig. 6a), and subsequent mass-spectroscopy (Fig. 6b) confirmed a molecular mass of 5664 (theoretical MW = 5663). These data confirm that we have obtained pure (> 90%) and correctly folded B22D desB30 insulin analog through recombinant expression and purification. Table 2 summarizes total yield and purity of our DPIP purification.

**Table 2:** Buffers used in protein purification

Buffers	Details
Buffer A	50 mM Tris-HCl, 2 mM EDTA, 100 mM NaCl
Buffer B	50 mM Tris-HCl, 1 mM EDTA, 100 mM NaCl,1% Tween
Buffer C	50 mM Tris-HCl, 1 mM EDTA, 100 mM NaCl, 1% Tween, 4 M Urea
Buffer D	50 mM Tris-HCl, 1 mM EDTA, 100 mM NaCl, 3% Tween
Buffer E	50 mM Tris-HCl, 1 mM EDTA, 100 mM NaCl,0.5% Tween
Buffer F	50 mM Tris-HCl, 1 mM EDTA, 10 mM β- Mercaptoethanol, 2 mM Sodium deoxycholate pH 8.0, 8 M Urea
TGE buffer	50 mM Tris-HCl, 0.5 mM EDTA,50 mM NaCl, 5% Glycerol, 1% Glycine, 0.1% Glutathione pH 8.0
Digestion buffer	20 mM Tris–HCl, 200 mM NaCl and 1 mM EDTA, pH 8.0
Buffer G	0.1% TFA
Buffer H	90% HPLC grade acetonitrile in H_2_O, 0.08% TFA

**Fig. 7:**
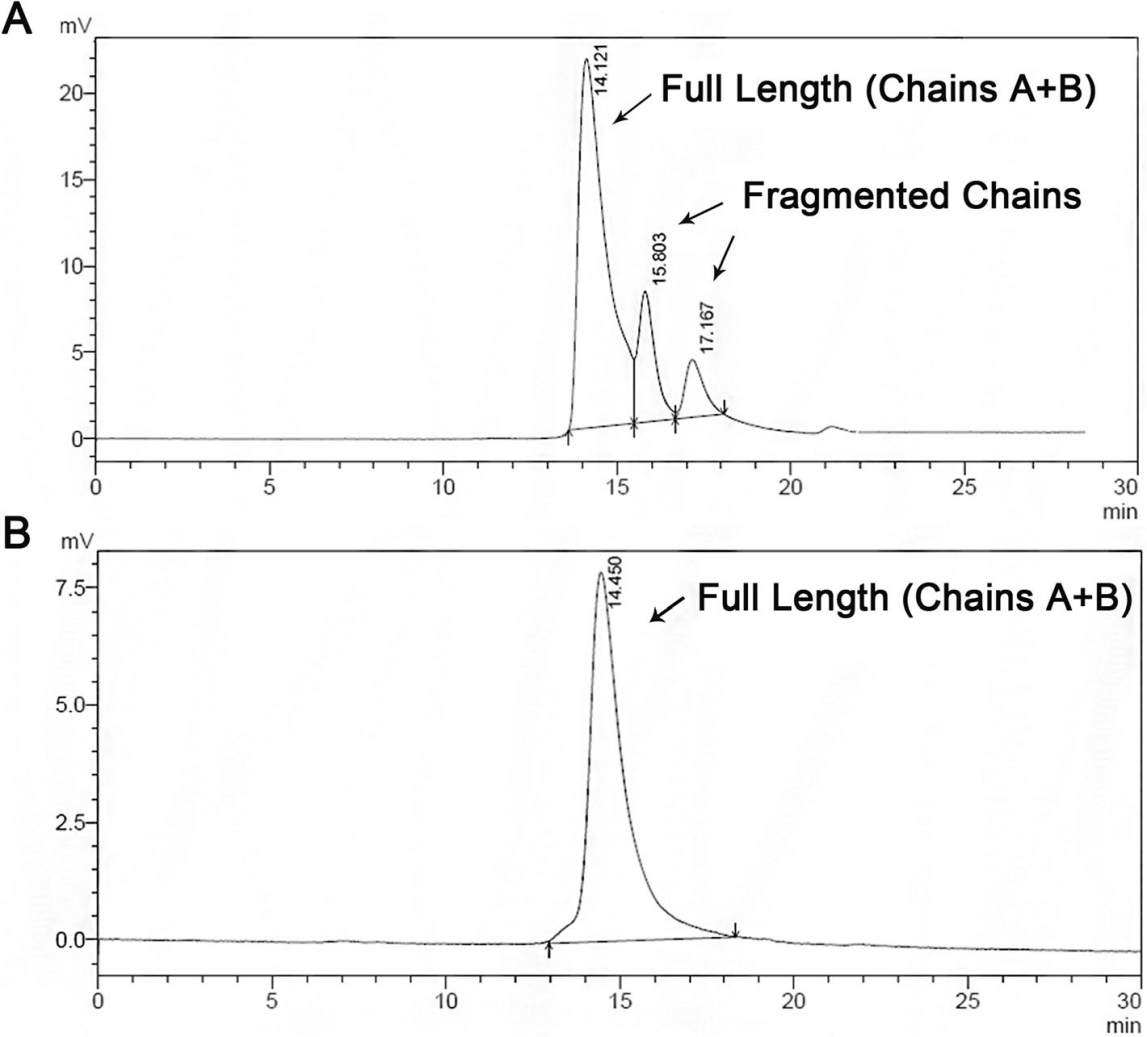
SEC elution profile of B22D desb30 insulin compared with commercial sample. Size exclusion chromatography of **a**): commercially available insulin sample and **b**). B22D desB30 insulin. The UV absorption was detected at 280 nm. Column: GE Superdex 75 Flow rate: 0.5 ml/min. FPLC: GE Healthcare Purifier 100

We further determined the quality and monomeric state of the B22D desB30 insulin by comparing our sample with commercially available monomeric recombinant insulin through size exclusion chromatography (Fig. 7). The recombinant B22D desB30 insulin eluted as a single peak at 15 min, which coincides with the major peak in the commercial sample, confirming the purity and monomeric nature of the B22D desB30 analog. Moreover, our sample showed no detectable degradation or fragmentation (Fig. 7b). Since the target peptide remains stable, we recommend using large-scale SEC to separate it for mass production.

Since misformed disulfides will induce lower solubility [25–27], faster degradation [28, 29], and unwanted aggregation or precipitation [30, 31], our final product displayed excellent solubility and superior stability, compared to commercially available insulin (Fig. 7), indicating no evidence of any misformed disulfides. This result also agrees with previous studies [32, 33] where similar recombinantly expressed proinsulin all folded correctly.

## Conclusion

This study aims to explore a cheaper alternative for producing monomeric human insulin analog to treat diabetes. Compared to the chemical synthesis and yeast preparation, our recombinant monomeric B22Asp desB30 insulin analog is fused to an N-terminal chitin-binding domain and intein self-cleavage tag. The fusion protein binds to chitin resins and is subsequently washed off. Next, trypsin digest releases mature insulin. The resulting product appears monomeric, > 90% pure and devoid of any exogenous enzyme. Thus, biologically active insulin analog can be efficiently produced in bacteria and potentially applicable in the treatment of human diabetes.

## Methods

All the buffers used in this study are summarized in Table 2.

### Plasmid construction

The coding sequence of the insulin precursor (PIP) was used as a template for subsequent site-directed-mutagenesis amplification (B22D) with Pfu under standard conditions using the primer pair M1/M2 (sequence: 5′- TTGGTCTGTGGTGAAGACGGTTTCTTCTACACC-3′ and 5′-GGTGTAGAAGAAACCGTCTTCACCACAGACCAA-3′). The coding sequence of B22D desB30 was flanked by 5′ NcoI and 3′ PstI restriction sites for inserting into the pTWIN1 (New England Biolabs) expression vector with the primer pair P1/P2 (sequence: 5′-ATATCCATGGGC AAGTTCGTCAACCAACA-3′ and 5′-ATATCCATGGGCAAGTTCGTCAA CCAACA-3′). P2 inserted an additional lysine residue at the N-terminus of DPIP to introduce a trypsin cleavage site. The recombinant plasmid was subsequently transformed into *E. coli* DH5α cells and confirmed by colony PCR and sequencing (Invitrogen Biotechnology Co., Ltd.). Sequences of the primers for colony PCR were primer F and R (5′-ACTGGGACTCCATCGTTTCT-3′and 5′-ATATCTGCAGCTAGTTACAGTAGTTCT-3′). The sequence-confirmed recombinant plasmid was named pTWIN1-DPIP. The design of the recombinant protein and purification scheme are illustrated in Fig. 1.

### Protein expression

All expression experiments were performed in duplicate with good reproducibility. First, pTWIN1-DPIP were transformed into *E. coli* strain BL21 (DE3) and grown to optical density (A600) of 0.5, in 300 ml of LB media (2% tryptone, 1% yeast extract, 2% NaCl, w/v) containing 100 μg/ml ampicillin at 37 °C. Next, isopropyl 1-thio-β-D-galactopyranoside (IPTG) was added to the final concentration of 0.1 mM to induce expression for 4 h at 37 °C. The cells were harvested by centrifugation at 3000 g for 15 min at 4 °C and stored at − 80 °C.

### Protein purification

All subsequent steps in this section were performed on ice or at 4 °C unless otherwise mentioned. Cell pellets were resuspended in ice-cold PBS (pH 8.0) at a ratio of 1:10 (w/v) and lysed by sonication. Since the DPIP is enriched in the insoluble fraction, the cell extracts were centrifuged at 10,000 g for 30 min at 4 °C to remove the soluble fraction. The pellets were washed sequentially with buffer A-E before redissolved thoroughly in ice-cold buffer F. Next, the mixture was dialyzed against TGE buffer (pH_8.0) with different concentrations of urea (6 M, 4 M, 2 M, 1 M, and 0 M). The insoluble fraction was removed by centrifugation at 10,000 g for 30 min.

Then the supernatant was applied to pre-equilibrated chitin affinity resins (resin diameter = 50–70 μm, New England Biolabs). The on-column cleavage of the intein fusion protein was conducted in 2 bed volumes of TGE buffer with a pH gradient (8.0, 7.5, 7.0, 6.5, 6.0, 5.5, 5.0, and 4.5) at desired temperatures (10 °C, 25 °C, 30 °C). The flow through was collected and analyzed by 12% SDS-PAGE and 16.5% Tricine -SDS-PAGE.

### Quantifying the intein splice efficiency

Premature cleavage was estimated by quantifying the scanned SDS polyacrylamide gels with Bandscan 5.0 (Glyko). Bands corresponding to full-length fusion protein (CBD-intein1-DPIP, CI-DPIP) and CBD-intein1 tag (CI) were quantified by scanning densitometry and normalized against their molecular weights.

### Optimizing tryptic digestion

The eluate from intein splicing was digested by trypsin at 30 °C for one hour to obtain the final B22D desB30 insulin analog. We used an enzyme/substrate ratio of 1:200 (w/w). The mixture was subsequently purified by high-pressure liquid chromatography (Wasters) (C8 column, acetonitrile and trifluoroacetic acid (TFA) as mobile phases). Monomeric B22D insulin analog was eluted at 19.5-min mark with a flow rate of 1 ml/min and a buffer H gradient of 35–50% over 30 min.

To access the quality of our sample, both the commercial insulin (Insulin Injection, MW 5778) and the B22D desB30 insulin analog was subjected to size exclusion chromatography (SEC) using Superdex 75 (GE healthcare) column under standard conditions.

## Data Availability

The datasets supporting the conclusions of this study are available upon request from the corresponding author.

## Abbreviations

B22D: B22Asp desB30 insulin
B22D-PIP/ DPIP: Insulin precursor of B22D
CBD: Chitin binding domain
CI: Chitin binding domain- intein1 tag
IPTG: Isopropyl 1-thio-β-D-galactopyranoside
SEC: Size exclusion chromatography
TFA: Trifluoroacetic acid
